# The Succinated Proteome of FH-Mutant Tumours

**DOI:** 10.3390/metabo4030640

**Published:** 2014-08-07

**Authors:** Ming Yang, Nicola Ternette, Huizhong Su, Raliat Dabiri, Benedikt M. Kessler, Julie Adam, Bin Tean Teh, Patrick J. Pollard

**Affiliations:** 1 Cancer Biology and Metabolism Group, Institute of Genetics and Molecular Medicine, Edinburgh Cancer Research UK Centre, University of Edinburgh, Western General Hospital, Crewe Road South, Edinburgh EH4 2XR, UK; E-Mails: ming.yang@igmm.ed.ac.uk (M.Y.); huizhong.su@igmm.ed.ac.uk (H.S.); rd292@exeter.ac.uk (R.D.); 2 Central Proteomics Facility, Target Discovery Institute, University of Oxford, Oxford OX3 7FZ, UK; E-Mails: nicola.ternette@ndm.ox.ac.uk (N.T.); benedikt.kessler@ndm.ox.ac.uk (B.M.K.); 3 Oxford Centre for Diabetes, Endocrinology & Metabolism, University of Oxford, Oxford OX3 7LJ, UK; E-Mail: julie.adam@ocdem.ox.ac.uk; 4 Cancer Science Institute of Singapore, National University of Singapore, Centre for Translational Medicine, #12-01, Singapore 117599; E-Mail: teh.bin.tean@singhealth.com.sg

**Keywords:** fumarate hydratase, succination, cysteine, renal cancer, hereditary leiomyomatosis and renal cell cancer (HLRCC), oncometabolite, biomarker, reactive oxygen species (ROS)

## Abstract

Inherited mutations in the Krebs cycle enzyme fumarate hydratase (FH) predispose to hereditary leiomyomatosis and renal cell cancer (HLRCC). Loss of FH activity in HLRCC tumours causes accumulation of the Krebs cycle intermediate fumarate to high levels, which may act as an oncometabolite through various, but not necessarily mutually exclusive, mechanisms. One such mechanism, succination, is an irreversible non-enzymatic modification of cysteine residues by fumarate, to form *S*-(2-succino)cysteine (2SC). Previous studies have demonstrated that succination of proteins including glyceraldehyde 3-phosphate dehydrogenase (GAPDH), kelch-like ECH-associated protein 1 (KEAP1) and mitochondrial aconitase (ACO2) can have profound effects on cellular metabolism. Furthermore, immunostaining for 2SC is a sensitive and specific biomarker for HLRCC tumours. Here, we performed a proteomic screen on an FH-mutant tumour and two HLRCC-derived cancer cell lines and identified 60 proteins where one or more cysteine residues were succinated; 10 of which were succinated at cysteine residues either predicted, or experimentally proven, to be functionally significant. Bioinformatic enrichment analyses identified most succinated targets to be involved in redox signaling. To our knowledge, this is the first proteomic-based succination screen performed in human tumours and cancer-derived cells and has identified novel 2SC targets that may be relevant to the pathogenesis of HLRCC.

## 1. Introduction

The accumulation of metabolites resulting from cancer-associated mutations in genes encoding key metabolic enzymes has been proposed to drive oncogenic transformation [1]. Mutations in genes encoding isocitrate dehydrogenase 1 and 2 (IDH1/2), succinate dehydrogenase (SDH) and fumarate hydratase (FH) can lead to high intracellular levels of d-2-hydroxyglutarate (D2HG), succinate and fumarate, respectively [2]. Due to their structural similarity to 2-oxoglutarate (2OG), these oncometabolites have been demonstrated to modulate the activities of 2OG-dependent dioxygenases; a family of enzymes with diverse functions including epigenetic regulation, oxygen sensing, collagen maturation and regulation of translation [3,4,5,6,7,8]. Fumarate is an electrophile that reacts with cysteine residues in susceptible proteins to form *S*-(2-succino)cysteine (2SC), a biomarker of mitochondrial stress in obesity and diabetes, and of FH-deficiency in hereditary leiomyomatosis and renal cell cancer (HLRCC) patients [9,10,11,12]. Succination is a non-enzymatic and irreversible reaction, which seems to preferentially target cysteine residues with low pKa values [13], although other factors affecting the susceptibility to succination may exist. To date, several studies have detected succinated proteins in a variety of animal and cellular models and have identified functional consequences associated with a range of cellular responses including activation of the Nuclear factor (erythroid-derived 2)-like 2 (NRF2)-mediated antioxidant pathway, inhibition of glyceraldehyde 3-phosphate dehydrogenase (GAPDH) and mitochondrial aconitase (ACO2) activity, and amplification of reactive oxygen species (ROS) signaling [13,14,15,16]. Here, we performed a proteomic-based screen using tandem mass spectrometry (MS/MS) to analyse an FH-deficient tumour and two FH-mutant cell lines derived from metastatic renal cancers. We identified 60 succinated proteins, 10 of which were modified at cysteine residues either predicted or experimentally proven to be functionally important. This study has expanded our knowledge of the succinated proteome in FH-associated renal cancer and identified novel 2SC targets, which may contribute to HLRCC tumourigenesis.

## 2. Results and Discussion

### 2.1. HLRCC Tumours and Derived Cell Lines Exhibit High Levels of 2SC

**Figure 1:**
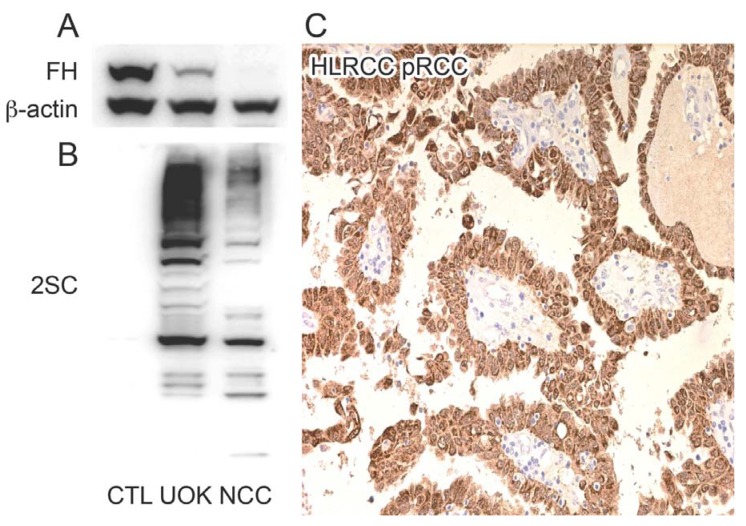
Hereditary leiomyomatosis and renal cell cancer (HLRCC) tumours and tumour-derived cell lines express high levels of 2SC. (**A**) Immunoblotting confirms the presence of fumarate hydratase (FH) in the control human renal proximal tubular epithelial (RPTC) cell line (CTL), the presence of the mutant FH allele in the UOK262 (UOK) HLRCC-derived cell line as previously described and absence of FH protein in the NCC-FH-1 (NCC) HLRCC-derived cell line. β-actin levels were used as a loading control for each cell line. (**B**) Immunoblotting for 2SC clearly shows an absence of 2SC expression in the control cell line but high expression of 2SC in the FH-mutant cell lines (UOK and NCC). (**C**) Positive 2SC immunoreactivity (indicated by brown staining) is evident in the tumour cells of an FH mutant type 2 PRCC (HLRCC PRCC) but absent in the stromal tissue (blue staining).

In order to verify the presence of succinated proteins in the FH-mutant tumour-derived cell lines UOK262 [17] and NCC-FH-1 (unpublished cell line kindly provided by Prof. Bin Teh), we performed immunoblotting using an anti-2SC antibody [18]. Strong immunoreactivity for 2SC was observed in both of these HLRCC cell lines but was absent in a normal renal proximal tubular epithelial cell line (RPTC) [19,20] (Figure 1B). Similarly immunohistochemistry detected high levels of 2SC in an FH-mutant (HLRCC) type 2 PRCC though was absent in the stromal tissue (Figure 1C) and sporadic type 2 PRCC (data not shown and as previously described [11]). We therefore performed a proteomic screen to identify succinated proteins in the cell lines and HLRCC tumour described above.

### 2.2. Multiple Proteins Are Succinated in FH-Mutant Cells and Tumours

As previously reported, 2SC modification is very specific to cells with elevated fumarate [11,21,22] and we did not detect this modification in FH wildtype RPTC cells or tumour stromal tissue cells by immunoblotting and immunohistochemistry respectively (Figure 1). However, in the two HLRCC-derived cell lines and tumour, succinated peptides (as determined by a 116 Da mass shift [11]) were detected in 60 different proteins (Table 1). Furthermore, 10 of these proteins were succinated on cysteine residues with potentially regulatory functions (Table 1; red font).

**Table 1:** Succinated proteins in HLRCC cancers and derived cell lines.

Uniprot ID	Protein Symbol	Protein Name	Succination Site	Source
P53396	ACLY	ATP-citrate synthase	C20	T, N
Q09666	AHNAK	Neuroblast differentiation-associated protein AHNAK	C1833	T, N
P15121	AKR1B1	Aldose reductase	C299	T
P54886-1	ALDH18A1	Isoform Long of Delta-1-pyrroline-5-carboxylate synthase	C612	T, U, N
Q5TYW2	ANKRD20A1	Ankyrin repeat domain-containing protein 20A1	C789	N
P04083	ANXA1	Annexin A1	C324	T, U
P16615-1	ATP2A2	Isoform 1 of Sarcoplasmicendoplasmic reticulum calcium ATPase 2	C997	N
Q86VP6-1	CAND1	Isoform 1 of Cullin-associated NEDD8-dissociated protein 1	C942	U
P20810-2	CAST	Isoform 2 of Calpastatin	C408, C413	N
P23528	CFL1	Cofilin-1	C139	T, U
Q9NX63	CHCHD3	Coiled-coil-helix-coiled-coil-helix domain-containing protein 3, mitochondrial	C112	T
Q8N5K1	CISD2	CDGSH iron-sulfur domain-containing protein 2	C92	T
Q00610-1	CLTC	Isoform 1 of Clathrin heavy chain 1	C870	U, N
P55060-1	CSE1L	Isoform 1 of Exportin-2	C272	U, N
Q6NSH3	CT45A5	Cancertestis antigen family 45 member A5	C22	U
Q9UBR2	CTSZ	Cathepsin Z	C92	U
Q9H773	DCTPP1	dCTP pyrophosphatase 1	C162	T
P17844	DDX5	Probable ATP-dependent RNA helicase DDX5	C200	U
P33316-2	DUT	Isoform 2 of Deoxyuridine 5'-triphosphate nucleotidohydrolase, mitochondrial	C3	N
P07814	EPRS	Bifunctional aminoacyl-tRNA synthetase	C105	T, U
O95571	ETHE1	Protein ETHE1, mitochondrial	C170	T, U, N
P21333-2	FLNA	Isoform 2 of Filamin-A	C717, C2543	T, U, N
O75369-1	FLNB	Isoform 1 of Filamin-B	C2501	N
Q9HA64	FN3KRP	Ketosamine-3-kinase	C24	T
P02794	FTH1	Ferritin heavy chain	C91	T
P04406	GAPDH	Glyceraldehyde-3-phosphate dehydrogenase	C152	T, U, N
P07203	GPX1	Glutathione peroxidase 1	C202	T
P53701	HCCS	Cytochrome c-type heme lyase	C39	U, N
P00492	HPRT1	Hypoxanthine-guanine phosphoribosyltransferase	C106	T
Q14197	ICT1	Peptidyl-tRNA hydrolase ICT1, mitochondrial	C82	N
Q9NWZ3	IRAK4	Isoform 1 of Interleukin-1 receptor-associated kinase 4	C13	U, N
O14880	MGST3	Microsomal glutathione *S*-transferase 3	C150, C151	T, N
P46013-1	MKI67	Isoform Long of Antigen KI-67	C1285	U
P35579-1	MYH9	Isoform 1 of Myosin-9	C988	T, U, N
Q9NX24	NHP2	HACA ribonucleoprotein complex subunit 2	C18	T
P53384-1	NUBP1	Isoform 1 of Cytosolic Fe-S cluster assembly factor NUBP1	C22, C25	T
Q9H6K4-1	OPA3	Isoform 1 of Optic atrophy 3 protein	C164	N
Q99497	PARK7	Protein DJ-1	C106	T, N
Q9Y570-1	PPME1	Isoform 1 of Protein phosphatase methylesterase 1	C381	N
Q06830	PRDX1	Peroxiredoxin-1	C173	T
P30048	PRDX3	Thioredoxin-dependent peroxide reductase, mitochondrial	C108	T
P30041	PRDX6	Peroxiredoxin-6	C91	T, U, N
Q15185	PTGES3	Prostaglandin E synthase 3	C58	T, U, N
P49023-2	PXN	Isoform Alpha of Paxillin	C535, C538	U
P63000-1	RAC1	Isoform A of Ras-related C3 botulinum toxin substrate 1	C178	T
P54727	RAD23B	UV excision repair protein RAD23 homolog B	C390	U
P50914	RPL14	Ribosomal protein L14 variant	C42	T
P05386	RPLP1	60S acidic ribosomal protein P1	C61	T
P31947-1	SFN	Isoform 1 of 14-3-3 protein sigma	C38	T, U, N
Q15005	SPCS2	Signal peptidase complex subunit 2	C17, C26	T, U, N
P42224-1	STAT1	Isoform Alpha of Signal transducer and activator of transcription 1-alphabeta	C492	N
Q00059	TFAM	Transcription factor A, mitochondrial	C246	T, N
Q12931	TRAP1	Heat shock protein 75 kDa, mitochondrial	C573	N
Q9H4B7	TUBB1	Tubulin beta-1 chain	C12	T, U, N
P10599	TXN	Thioredoxin	C73	T
P09936	UCHL1	Ubiquitin carboxyl-terminal hydrolase isozyme L1	C90 (T), C152 (T, U)	T, U
P45880-2	VDAC2	Isoform 2 of Voltage-dependent anion-selective channel protein 2	C76	T, U, N
Q9Y277-1	VDAC3	Isoform 1 of Voltage-dependent anion-selective channel protein 3	C65	T, U, N
P08670	VIM	Vimentin	C328	T
P54577	YARS	Tyrosyl-tRNA synthetase, cytoplasmic	C424	U

2SC targets identified an HLRCC tumour and tumour-derived cell lines. Succinated proteins are listed alphabetically by gene symbol, with succinated cysteine residues indicated. Targets highlighted in red indicate cysteine residues with either predicted or experimentally proven roles in protein function [23]. T = HLRCC tumour, U = UOK262 cell line, N = NCC-FH-1 cell line.

### 2.3. Succination Preferentially Targets Proteins Involved in Redox Regulation

**Figure 2:**
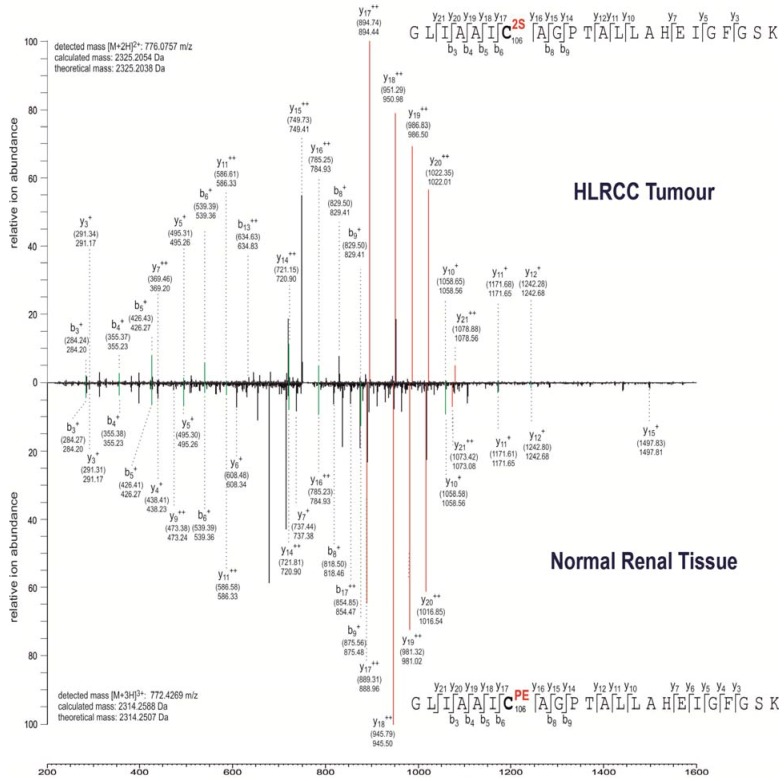
MS/MS spectra showing succination of the active site (C106) of PARK7/DJ-1.

**Figure 3:**
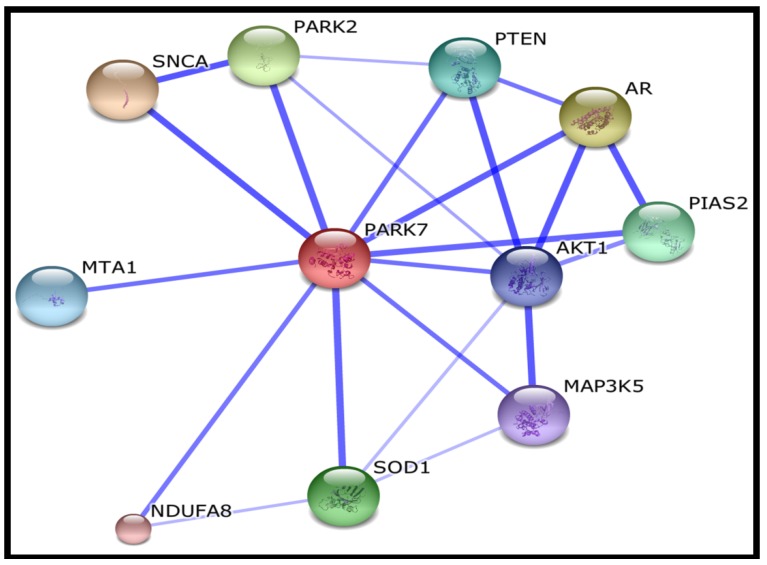
Protein interactions of the redox-sensitive succination target PARK7/DJ-1.

Hierarchical clustering and gene ontology analyses suggest that most of the succination targets identified are involved in redox homeostasis or subject to ROS regulation (Table 2). This is unsurprising considering the critical roles of cysteine residues in cellular oxidative stress regulation (reviewed in [24]). Previous studies have demonstrated the functional consequences of protein succination, including inhibition of the metabolic enzymes GAPDH and ACO2 [15,16], loss of KEAP1 function resulting in constitutive NRF2 expression in type 2 papillary RCC (PRCC) and Fh1-deficient mice [25,26] and modification of reduced glutathione (GSH) in FH-deficient cells resulting in the amplification of ROS-dependent signaling [14]. In this study, we identified succination of Cys-106 at the active site of Parkinson disease protein 7 (PARK7) (also known as protein DJ-1) (Figure 2), a redox-sensitive protein that is implicated in cellular protection against oxidative stress in Parkinson’s disease (reviewed in [27]), mediates cellular responses to hypoxia [28] and can regulate metabolic pathways in RCC [29]. Interestingly PARK7 is closely associated with NRF2 through interaction with Superoxide dismutase 1 (SOD1) [30] (Figure 3). Further, NRF2 and PARK7 are prognostic factors in lung cancer and upregulated in inflammatory multiple sclerosis lesions [31,32]. Other succinated cysteine residues that may be critical to protein functions (highlighted in red font in Table 1) include Cys-299 of Aldose reductase (AKR1B1), which catalyzes the NADPH-dependent reduction of a wide variety of carbonyl-containing compounds to their corresponding alcohols with a broad range of catalytic efficiencies. Evidence suggests that Cys-299 may regulate the kinetic and inhibition properties of AKR1B1, but does not participate in catalysis [33]. Peroxiredoxins are antioxidant enzymes that control cytokine-induced peroxide levels and thereby mediate signal transduction in mammalian cells [34]. We observed succination of peroxiredoxin 1 and 3 (PRDX1 and PRDX3) at Cys-173 and Cys-108, respectively, both of which participate in intermolecular disulfide formation and are crucial to their molecular function [34,35]. Succination was also detected in thioredoxin at a crucial residue Cys-73, which acts as a donor for nitrosylation of target proteins and therefore its succination may potentially impair the *S*-nitrosylating activity of thioredoxin [36]. Isoform 1 of Cytosolic Fe-S cluster assembly factor Nucleotide binding protein 1 (NUBP1) is succinated at residues Cys-22 and Cys-25, both of which are involved in iron-sulfur cluster (4Fe-4S) assembly [37]. Succination of NUBP1 may lead to enzymatic inhibition, similar to that observed in ACO2 [15]. Further, microsomal glutathione *S*-transferase 3 (MGST3) is succinated at Cys-150 and Cys-151, both of which are also targets of *S*-palmitoylation [38]. Site-directed mutagenesis of cysteines for some of the above proteins provides experimental evidence for their functional roles (Table 3). Therefore modification by succination may be predicted to cause either a loss or gain of function in these proteins.

## 3. Experimental Section

### 3.1. Cell Lines and Human Tissue Samples

Cell lines were cultured as previously described [39]. The UOK262 cell line was a kind gift from Dr Marston Linehan [17], and the NCC-FH-1 cell line from Professor Bin Teh (characterized by Choon Kiat Ong, Min Han Tan, Bernice Wong and Victoria Perrier-Trudova). The RPTC cell line was a kind gift from Professors Albert Ong and Lorraine Racusen. Anonymized human tumour and normal samples were collected with full ethical approval (MREC 05/Q1605/66) as approved by the Oxford Centre for Histopathology Research.

**Table 2:** Gene ontology analyses of succinated proteins.

Category	Term	Count	%	*p* Value
GOTERM_BP	GO:0000302~response to reactive oxygen species	7	11.67	3.50 × 10^−7^
GOTERM_BP	GO:0042542~response to hydrogen peroxide	6	10.00	2.06 × 10^−6^
GOTERM_BP	GO:0042743~hydrogen peroxide metabolic process	5	8.33	2.14 × 10^−6^
GOTERM_MF	GO:0051920~peroxiredoxin activity	4	6.67	3.35 × 10^−6^
GOTERM_BP	GO:0034614~cellular response to reactive oxygen species	5	8.33	4.57 × 10^−6^
GOTERM_MF	GO:0016684~oxidoreductase activity, acting on peroxide as acceptor	5	8.33	7.57 × 10^−6^
GOTERM_MF	GO:0004601~peroxidase activity	5	8.33	7.57 × 10^−6^
GOTERM_BP	GO:0034599~cellular response to oxidative stress	5	8.33	1.98 × 10^−5^
GOTERM_BP	GO:0042744~hydrogen peroxide catabolic process	4	6.67	3.31 × 10^−5^
GOTERM_BP	GO:0006979~response to oxidative stress	7	11.67	3.32 × 10^−5^
GOTERM_MF	GO:0016209~antioxidant activity	5	8.33	3.59 × 10^−5^
GOTERM_BP	GO:0070301~cellular response to hydrogen peroxide	4	6.67	3.96 × 10^−5^
GOTERM_BP	GO:0045454~cell redox homeostasis	5	8.33	9.06 × 10^−5^
GOTERM_BP	GO:0010035~response to inorganic substance	7	11.67	1.15 × 10^−4^
GOTERM_BP	GO:0006800~oxygen and reactive oxygen species metabolic process	5	8.33	1.15 × 10^−4^
GOTERM_BP	GO:0019725~cellular homeostasis	9	15.00	3.23 × 10^−4^
GOTERM_BP	GO:0042592~homeostatic process	11	18.33	4.20 × 10^−4^
GOTERM_BP	GO:0055114~oxidation reduction	10	16.67	5.67 × 10^−4^

Functional classification of succinated proteins using DAVID Bioinformatics Resources [40,41,42] ranked by statistical significance. Proteins were classified using the gene ontology functional annotation for biological processes (GOTERM_BP) and molecular function (GOTERM_MF). *p*-values < 0.001 were considered to be highly significant.

### 3.2. Proteomics and Mass Spectrometry

Tumour and normal kidney tissue and cells were homogenized and sonicated in Urea-SDS buffer and protein extracts separated by SDS-PAGE and processed for trypsin digestion and MS/MS analyses as previously described [15]. Database searches were performed against UniProt/SwissProt [23] or International Protein Index [43] database using Mascot [44] or CPFP 1.3.0 [45]. For label-free quantitation of succinated peptides, samples were analyzed in three technical replicates.

### 3.3. Immunohistochemistry (IHC) and Immunoblotting (IB)

Analyses of tumours and cell lines by IHC and IB were performed as previously described [11,46]. The 2SC antibody was a kind gift from Dr Norma Frizzell, the fumarase antibody from Nordic Labs (Copenhagen, Denmark), and the β-actin antibody purchased from Abcam (Cambridge, UK).

MS/MS spectra showing either succination (^2S^C) or pyridylethylation (^PE^C) at cysteine 106 in the _100_GLIAAICAGPTALLAHEIGFGSK_122_ peptide of human DJ-1 derived from human HLRCC tumour tissue and normal renal tissue, respectively. Fragment ions are indicated for the peptide sequence and are labelled as follows: b: *N*-terminal fragment ion; y: *C*-terminal fragment ion; ^+^: singly charged fragment ion and ^++^: doubly charged fragment ion. Both theoretical mass and detected mass (in brackets) are given for each assigned fragment ion. Matching fragment ion peaks between the two peptide species that do not contain the modified residue are highlighted in green, whereas peptide fragments of different mass that contain the modified residue are highlighted in red.

PARK7 acts as a positive regulator of androgen receptor- dependent transcription, prevents aggregation of SNCA, protects neurons against oxidative stress and cell death and potentially functions as a redox-sensitive chaperone and as a sensor for oxidative stress. Figure generated by STRING v9.1 [47,48].


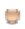

SNCA
synuclein, alpha (non A4 component of amyloid precursor)
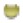

AR
androgen receptor
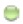

PARK2
Parkinson disease (autosomal recessive, juvenile) 2, parkin
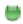

SOD1
superoxide dismutase 1, soluble
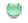

PIAS2
protein inhibitor of activated STAT, 2
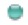

PTEN
phosphatase and tensin homolog; Tumour suppressor
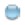

MTA1
metastasis associated 1
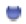

AKT1
v-akt murine thymoma viral oncogene homolog 1
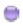

MAP3K5
mitogen-activated protein kinase kinase kinase 5
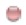

NDUFA8
NADH dehydrogenase (ubiquinone) 1 alpha subcomplex, 8

### 3.4. Gene Ontology Analysis

Functional classification of succinated proteins was performed using DAVID Bioinformatics Resources v6.7 (National Institute of Allergy and Infectious Diseases (NIAID), NIH, Frederick, MD, USA) [41,42]. Protein network analysis for PARK7 was generated using STRING v9.1 [47,48].

**Table 3:** Mutational studies on cysteine residues observed to be succinated in FH-mutant tumours and cancer cells.

Uniprot ID	Protein Symbol	Succination Site (S)	Mutational Data	References
O14880	GAPDH	C150, C151	C → S: Abolishes *S*-acylation; when associated with S-151. C → S: Abolishes *S*-acylation; when associated with S-150	[38]
Q99497	PARK7	C106	C → A: Abolishes oxidation, association with mitochondria and protease activity. No effect on chaperone activity. Reduced binding to OTUD7B. C → A: Reduced localization in lipid rafts; when associated with A-46. C → D: Abolishes oxidation and association with mitochondria. No effect on chaperone activity. C → S: No effect on mitochondrial translocation. Reduced protease activity.	[49,50,51,52,53,54,55]
P10599	TXN	C73	C → D: Strongly reduced *S*-nitrosylation of CASP3. C → S: Loss of nitrosylation, and loss of *S*-nitrosylating activity towards CASP3. Retains interaction with APEX1 and transcription activation; when associated with S-62 and S-69. C → S: Retains its reducing activity.	[36,56,57]
P09936	UCHL1	C90	C → S: Abolishes enzymatic activity.	[58,59,60]

Site-directed mutagenesis of cysteine residues that are targeted for succination, has provided evidence of their functional roles, which may be affected by succination.

## 4. Conclusions

Recent technological advances in mass spectrometry have facilitated high throughput screening of post-translational modifications in proteins. Here, we analysed cell lines and a tumour derived from HLRCC patients to try to identify the scope of the succinated proteome under FH-deficient settings. Succination of cysteine residues is a candidate mechanism for FH-associated oncogenesis, which is corroborated by genetic and biochemical evidence generated from the analyses of Fh1 knockout mice and gene expression profiling and exome sequencing of type 2 RCC [25,26,61]. Interestingly, some peptides were detected in only one or two of the three FH-mutant samples. However, this is most probably due to the heterogeneity of RCC [62,63]; both the UOK262 and NCC-FH-1 cell lines were generated from RCC metastases from different patients ([17] and personal communication with Dr Teh). Furthermore, due to lack of antibody-based enrichment technologies, the “succinome” thus identified is biased towards abundant proteins in a cell line specific manner. Therefore we cannot rule out the possibility that the same succinated proteins were present in all samples, albeit at different levels, some below the threshold of detection. Given that 2SC was originally identified in diabetic rats and induced as a marker of mitochondrial stress in adipocytes resulting from glucotoxicity [12,16,18,64], it is likely that succination resulting from elevated levels of intracellular fumarate targets proteins involved in a diverse range of cellular and biological processes, including microtubule dynamics [65], the NRF2-mediated antioxidant response [24,25], altered metabolism [15,16,39] and as identified here, redox homeostasis. Further studies are required to test the functionality and biological consequences of these modifications.
